# The γ-polymorph of AgZnPO_4_ with an ABW zeolite-type framework topology

**DOI:** 10.1107/S1600536810038717

**Published:** 2010-10-02

**Authors:** Abderrazzak Assani, Mohamed Saadi, Lahcen El Ammari

**Affiliations:** a Laboratoire de Chimie du Solide Appliquée, Faculté des Sciences, Université Mohammed V-Agdal, Avenue Ibn Battouta, BP 1014, Rabat, Morocco

## Abstract

The γ-polymorph of the title compound, silver zinc orthophos­phate, was synthesized under hydro­thermal conditions. The structure consists of ZnO_4_, PO_4_ and AgO_4_ units. The coord­ination spheres of Zn^II^ and P^V^ are tetra­hedral, whereas the Ag^I^ atom is considerably distorted from a tetra­hedral coordination. Each O atom is linked to each of the three cations. An elliptic eight-membered ring system is formed by corner-sharing of alternating PO_4_ and ZnO_4_ tetra­hedra, leading to a framework with an ABW-type zeolite structure. The framework encloses channels running parallel to [100] in which the Ag cations are located, with Ag⋯Ag contacts of 3.099 (3) Å. This short distance results from *d* ^10^⋯*d* ^10^ inter­actions, which play a substantial role in the crystal packing. The structure of γ-AgZnPO_4_ is distinct from the two other polymorphs α-AgZnPO_4_ and β-AgZnPO_4_, but is isotypic with NaZnPO_4_-ABW, NaCoPO_4_-ABW and NH_4_CoPO_4_-ABW.

## Related literature

For general background to *A* ^I^ *B* ^II^PO_4_ phosphates, see: Elouadi & Elammari (1990 ▶); Bu *et al.* (1996 ▶); Moring & Kostiner (1986 ▶). For the α- and β- polymorphs of AgZnPO_4_, see: Hammond *et al.* (1998 ▶); Elammari *et al.* (1987 ▶, 1988 ▶). For bond-valence analysis, see: Brown & Altermatt (1985 ▶). For *d* ^10^⋯*d* ^10^ inter­actions, see: Jansen (1987 ▶). For compounds with isotypic structures, see: Chippindale *et al.* (1999 ▶); Feng *et al.* (1997 ▶); Ng & Harrison (1998 ▶). For nomenclature of zeolites, see: Baerlocher *et al.* (2007 ▶).

## Experimental

### 

#### Crystal data

AgZn(PO_4_)
*M* *_r_* = 268.21
Monoclinic, 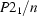

*a* = 5.1664 (2) Å
*b* = 10.4183 (3) Å
*c* = 7.3263 (2) Å
β = 90.304 (2)°
*V* = 394.33 (2) Å^3^
*Z* = 4
Mo *K*α radiation
μ = 11.32 mm^−1^
*T* = 296 K
0.25 × 0.08 × 0.05 mm

#### Data collection

Bruker X8 APEXII diffractometer
Absorption correction: multi-scan (*SADABS*; Bruker, 2005 ▶) *T*_min_ = 0.351, *T*_max_ = 0.568
9307 measured reflections
1745 independent reflections
1621 reflections with *I* > 2σ(*I*)
*R*_int_ = 0.033

#### Refinement

*R*[*F* ^2^ > 2σ(*F* ^2^)] = 0.019
*wR*(*F* ^2^) = 0.044
*S* = 1.08
1745 reflections
65 parameters
Δρ_max_ = 1.18 e Å^−3^
Δρ_min_ = −1.30 e Å^−3^

### 

Data collection: *APEX2* (Bruker, 2005 ▶); cell refinement: *SAINT* (Bruker, 2005 ▶); data reduction: *SAINT*; program(s) used to solve structure: *SHELXS97* (Sheldrick, 2008 ▶); program(s) used to refine structure: *SHELXL97* (Sheldrick, 2008 ▶); molecular graphics: *ORTEP-3 for Windows* (Farrugia, 1997 ▶) and *DIAMOND* (Brandenburg, 2006 ▶); software used to prepare material for publication: *WinGX* (Farrugia, 1999 ▶).

## Supplementary Material

Crystal structure: contains datablocks I, global. DOI: 10.1107/S1600536810038717/wm2407sup1.cif

Structure factors: contains datablocks I. DOI: 10.1107/S1600536810038717/wm2407Isup2.hkl

Additional supplementary materials:  crystallographic information; 3D view; checkCIF report

## Figures and Tables

**Table 1:** Selected bond lengths (Å)

Ag1—O2^i^	2.2992 (13)
Ag1—O1	2.3506 (13)
Ag1—O4^ii^	2.3982 (13)
Ag1—O3^iii^	2.4975 (14)
Zn1—O1	1.9372 (13)
Zn1—O2^iv^	1.9439 (13)
Zn1—O3^ii^	1.9440 (13)
Zn1—O4^v^	1.9516 (13)
P1—O3	1.5283 (14)
P1—O1	1.5366 (13)
P1—O2	1.5401 (13)
P1—O4	1.5415 (13)

Symmetry codes: (i) 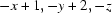; (ii) 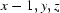; (iii) 
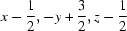
; (iv) 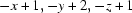; (v) 
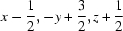
.

**Fig. 1.**
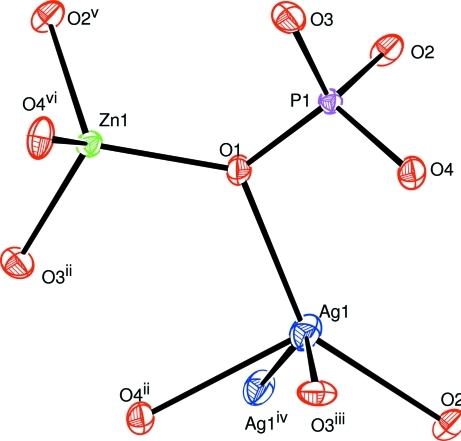
Plot of parts of the crystal structure of the title compound showing the most important interatomic bonds. Displacement ellipsoids are drawn at the 50% probability level. [Symmetry codes: (i) -x + 1, -y + 2, -z; (ii) x - 1, y, z; (iii) x - 1/2, -y + 3/2, z - 1/2; (iv) -x, -y + 2, -z; (v) -x + 1, -y + 2, -z + 1; (vi) x - 1/2, -y + 3/2, z + 1/2.]

**Fig. 2.**
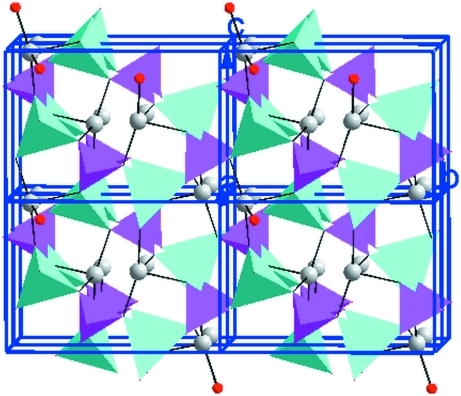
A three-dimensional polyhedral view of the crystal structure of the monophosphate γ-AgZnPO4. PO4 tetrahedra are pink, ZnO4 tetrahedra are light-blue and silver atoms are grey.

## supplementary crystallographic information

### Comment

A crystal-chemical classification of *A*^I^*B*^II^PO_4_ compounds was carried out by Elouadi and Elammari (1990) who used the combination of the coordination number and the correlative cationic radii *r*(*A*) and *r*(*B*) as basic parameters to predict the structural evolution *versus* the nature of both *A* and *B* elements. In fact, the appearance of a structural variety does not depend only on the size and the nature of both cations *A* and *B*, but could also be favored by specific parameters such as the Jahn-Teller effect, which mainly characterizes compounds containing Cu(II). In addition, the structural stability is also expected to be both temperature- and pressure-dependent. Therefore, the thermodynamic conditions for the preparation of all phases considered are of prime importance. This is also corroborated by the fact that most of the compounds *A*^I^*B*^II^PO_4_ undergo at least one phase transition (Elammari *et al.*, 1988). For instance, it has been found that the thermal treatment (quenching, sintering, *etc*.) is a key parameter to foresee the structural variety to be stabilized at room temperature (Moring & Kostiner 1986; Bu *et al.*, 1996). In addition to α-AgZnPO_4_ and β-AgZnPO_4_ characterized by Hammond *et al.*(1998), we report here on the crystal structure of a new form of silver zinc phosphate (γ-AgZnPO_4_) that was hydrothermally synthesized.

The structure of this monophosphate consists of zinc and phosphorus atoms tetrahedrally coordinated to oxygen atoms, whereas the silver atom is surrounded by four O atoms in a considerably distorted coordination, with Ag–O bond lengths between 2.2992 (13) and 2.4975 (14) Å. As shown in Fig. 1, the PO_4_ and ZnO_4_ tetrahedra share a vertex and are almost regular with P–O and Zn–O distances in the range 1.5283 (14)–1.5415 (13) Å and 1.9372 (13)–1.9516 (13) Å, respectively (Table 1). The expected +I, +II and +V oxidation states of the Ag, Zn and P atoms were confirmed by bond valence sum calculations (Brown & Altermatt, 1985) with 0.94, 2.09 and 4.93 valence units, respectively.

A three-dimensional polyhedral view of the crystal structure is represented in Fig. 2. It shows PO_4_ tetrahedra linked to ZnO_4_ tetrahedra by sharing corners in the way to build an eight-membered ring system surrounding the silver atoms. This arrangements give rise to eight-membered elliptical channels running parallel to [100] where the Ag^I^ atoms are located with short Ag···Ag contacts of 3.099 (3) Å. This short distance is due to *d*^10^···*d*^10^ interactions (Jansen, 1987) that play an important role in the crystal structure.

It is particularly interesting to compare the crystal structures of the three different polymorphs of AgZnPO_4_: The high-temperature β-AgZnPO_4_ polymorph adopts a monoclinic beryllonite-type structure similar to that of NaZnPO_4_ (Elammari *et al.*, 1987) whereas the low-temperature α-AgZnPO_4_ polymorph crystallizes with a hexagonal structure like that of high-*p*/low-*T* KZnPO_4_. In both α- and β- polymorphs, corner-sharing PO_4_ and ZnO_4_ tetrahedra form a fully ordered framework containing six-membered rings with distinct topologies around the Ag^I^ atoms (Hammond *et al.*, 1998). As noted above, in the case of γ-AgZnPO_4_ they build up an elliptical eight-membered ring system of alternating PO_4_ and ZnO_4_ tetrahedra around the Ag^I^ atoms with an ABW zeolite topology UUUUDDDD, where U and D represent tetrahedra pointing up and down, respectively (Baerlocher *et al.*, 2007).

Compounds isotypic with γ-AgZnPO_4_ are relatively rare, however, there are three phases which adopt the same stucture, *viz*. NaZnPO_4_-ABW (Ng & Harrison, 1998), NaCoPO_4_-ABW (Chippindale *et al.*, 1999) and NH_4_CoPO_4_-ABW (Feng *et al.*, 1997).

### Experimental

The hydrothermal exploration of the Ag_2_O–ZnO–P_2_O_5_ system, in order to search for new phases, in particular with alluaudite-like structure, has allowed to isolate a new form of silver zinc orthophosphate. The reaction mixture contained silver nitrate (AgNO_3_; 0.1699 g), zinc oxide (ZnO; 0.1221 g), 85 %_wt_ phosphoric acid (H_3_PO_4_; 0,10 ml) and water (12 ml) and was hydrothermally treated in a 23 ml Teflon-lined autoclave under autogeneous pressure at 468 K for two days. After being filtered off, washed with deionized water and air dried, the reaction product consists of a white powder and some colorless parallelepipedic crystals corresponding to the title compound.

### Refinement

The highest peak and the deepest hole in the final Fourier map are 0.53 Å and 0.52 Å, respectively, from Ag1.

### Crystal data

**Table:**

AgZn(PO_4_)	*F*(000) = 496
*M**_r_* = 268.21	*D*_x_ = 4.518 Mg m^−^^3^
Monoclinic, *P*2_1_/*n*	Mo *K*α radiation, λ = 0.71073 Å
Hall symbol: -P 2yn	Cell parameters from 1745 reflections
*a* = 5.1664 (2) Å	θ = 3.4–35.0°
*b* = 10.4183 (3) Å	µ = 11.32 mm^−^^1^
*c* = 7.3263 (2) Å	*T* = 296 K
β = 90.304 (2)°	Plate, colourless
*V* = 394.33 (2) Å^3^	0.25 × 0.08 × 0.05 mm
*Z* = 4	

### Data collection

**Table:**

Bruker X8 APEXII diffractometer	1745 independent reflections
Radiation source: fine-focus sealed tube	1621 reflections with *I* > 2σ(*I*)
graphite	*R*_int_ = 0.033
φ and ω scans	θ_max_ = 35.0°, θ_min_ = 3.4°
Absorption correction: multi-scan (*SADABS*; Bruker, 2005)	*h* = −7→8
*T*_min_ = 0.351, *T*_max_ = 0.568	*k* = −16→16
9307 measured reflections	*l* = −11→11

### Refinement

**Table:**

Refinement on *F*^2^	Primary atom site location: structure-invariant direct methods
Least-squares matrix: full	Secondary atom site location: difference Fourier map
*R*[*F*^2^ > 2σ(*F*^2^)] = 0.019	*w* = 1/[σ^2^(*F*_o_^2^) + (0.0138*P*)^2^ + 0.5475*P*] where *P* = (*F*_o_^2^ + 2*F*_c_^2^)/3
*wR*(*F*^2^) = 0.044	(Δ/σ)_max_ = 0.001
*S* = 1.08	Δρ_max_ = 1.18 e Å^−^^3^
1745 reflections	Δρ_min_ = −1.30 e Å^−^^3^
65 parameters	Extinction correction: *SHELXL97* (Sheldrick, 2008), Fc^*^=kFc[1+0.001xFc^2^λ^3^/sin(2θ)]^-1/4^
0 restraints	Extinction coefficient: 0.0685 (12)

### Special details

**Table:**

Geometry. All e.s.d.'s (except the e.s.d. in the dihedral angle between two l.s. planes) are estimated using the full covariance matrix. The cell e.s.d.'s are taken into account individually in the estimation of e.s.d.'s in distances, angles and torsion angles; correlations between e.s.d.'s in cell parameters are only used when they are defined by crystal symmetry. An approximate (isotropic) treatment of cell e.s.d.'s is used for estimating e.s.d.'s involving l.s. planes.
Refinement. Refinement of *F*^2^ against ALL reflections. The weighted *R*-factor *wR* and goodness of fit *S* are based on *F*^2^, conventional *R*-factors *R* are based on *F*, with *F* set to zero for negative *F*^2^. The threshold expression of *F*^2^ > σ(*F*^2^) is used only for calculating *R*-factors(gt) *etc*. and is not relevant to the choice of reflections for refinement. *R*-factors based on *F*^2^ are statistically about twice as large as those based on *F*, and *R*- factors based on ALL data will be even larger.

### Fractional atomic coordinates and isotropic or equivalent isotropic displacement parameters (Å^2^)

**Table:**

	*x*	*y*	*z*	*U*_iso_*/*U*_eq_	
Ag1	0.20254 (3)	0.891281 (15)	0.021499 (19)	0.01931 (6)	
Zn1	0.19054 (4)	0.840764 (19)	0.52546 (3)	0.01037 (6)	
P1	0.69041 (8)	0.89550 (4)	0.29147 (6)	0.00877 (8)	
O1	0.3966 (2)	0.87815 (14)	0.31182 (18)	0.0159 (2)	
O2	0.7616 (3)	1.03856 (12)	0.27484 (18)	0.0161 (2)	
O3	0.8268 (3)	0.83575 (14)	0.45641 (19)	0.0166 (2)	
O4	0.7730 (3)	0.83197 (12)	0.11089 (18)	0.0155 (2)	

### Atomic displacement parameters (Å^2^)

**Table:**

	*U*^11^	*U*^22^	*U*^33^	*U*^12^	*U*^13^	*U*^23^
Ag1	0.02148 (8)	0.02283 (8)	0.01363 (8)	0.00195 (5)	0.00071 (5)	0.00377 (5)
Zn1	0.01202 (9)	0.00970 (9)	0.00939 (9)	−0.00034 (6)	−0.00004 (6)	0.00058 (6)
P1	0.00988 (16)	0.00839 (16)	0.00805 (16)	−0.00085 (12)	0.00051 (12)	0.00001 (12)
O1	0.0099 (5)	0.0272 (7)	0.0107 (5)	−0.0013 (5)	0.0007 (4)	0.0032 (5)
O2	0.0289 (7)	0.0083 (5)	0.0110 (5)	−0.0043 (5)	0.0007 (5)	−0.0006 (4)
O3	0.0139 (5)	0.0184 (6)	0.0176 (6)	−0.0014 (4)	−0.0044 (4)	0.0069 (5)
O4	0.0189 (6)	0.0130 (5)	0.0147 (6)	−0.0045 (4)	0.0059 (5)	−0.0055 (4)

### Geometric parameters (Å, °)

**Table:**

Ag1—O2^i^	2.2992 (13)	P1—O1	1.5366 (13)
Ag1—O1	2.3506 (13)	P1—O2	1.5401 (13)
Ag1—O4^ii^	2.3982 (13)	P1—O4	1.5415 (13)
Ag1—O3^iii^	2.4975 (14)	O2—Zn1^v^	1.9438 (13)
Ag1—Ag1^iv^	3.0990 (3)	O2—Ag1^i^	2.2992 (13)
Zn1—O1	1.9372 (13)	O3—Zn1^vii^	1.9440 (13)
Zn1—O2^v^	1.9439 (13)	O3—Ag1^viii^	2.4975 (14)
Zn1—O3^ii^	1.9440 (13)	O4—Zn1^ix^	1.9516 (13)
Zn1—O4^vi^	1.9516 (13)	O4—Ag1^vii^	2.3982 (13)
P1—O3	1.5283 (14)		
			
O2^i^—Ag1—O1	146.80 (5)	O3—P1—O2	110.33 (8)
O2^i^—Ag1—O4^ii^	114.78 (5)	O1—P1—O2	110.97 (8)
O1—Ag1—O4^ii^	97.40 (5)	O3—P1—O4	112.03 (8)
O2^i^—Ag1—O3^iii^	95.66 (5)	O1—P1—O4	108.10 (8)
O1—Ag1—O3^iii^	90.50 (5)	O2—P1—O4	106.28 (7)
O4^ii^—Ag1—O3^iii^	92.72 (5)	P1—O1—Zn1	130.50 (8)
O2^i^—Ag1—Ag1^iv^	74.30 (4)	P1—O1—Ag1	108.80 (7)
O1—Ag1—Ag1^iv^	114.78 (4)	Zn1—O1—Ag1	120.61 (6)
O4^ii^—Ag1—Ag1^iv^	65.84 (3)	P1—O2—Zn1^v^	126.57 (8)
O3^iii^—Ag1—Ag1^iv^	147.90 (3)	P1—O2—Ag1^i^	113.75 (7)
O1—Zn1—O2^v^	114.19 (6)	Zn1^v^—O2—Ag1^i^	119.64 (6)
O1—Zn1—O3^ii^	109.25 (6)	P1—O3—Zn1^vii^	129.49 (8)
O2^v^—Zn1—O3^ii^	109.40 (7)	P1—O3—Ag1^viii^	114.76 (7)
O1—Zn1—O4^vi^	108.94 (6)	Zn1^vii^—O3—Ag1^viii^	103.00 (6)
O2^v^—Zn1—O4^vi^	109.17 (6)	P1—O4—Zn1^ix^	127.61 (8)
O3^ii^—Zn1—O4^vi^	105.52 (6)	P1—O4—Ag1^vii^	112.61 (7)
O3—P1—O1	109.09 (8)	Zn1^ix^—O4—Ag1^vii^	110.53 (6)

Symmetry codes: (i) −*x*+1, −*y*+2, −*z*; (ii) *x*−1, *y*, *z*; (iii) *x*−1/2, −*y*+3/2, *z*−1/2; (iv) −*x*, −*y*+2, −*z*; (v) −*x*+1, −*y*+2, −*z*+1; (vi) *x*−1/2, −*y*+3/2, *z*+1/2; (vii) *x*+1, *y*, *z*; (viii) *x*+1/2, −*y*+3/2, *z*+1/2; (ix) *x*+1/2, −*y*+3/2, *z*−1/2.
